# Safety Evaluation of Chlorantraniliprole in Lychee Based on Residue and Dietary Risk Assessment

**DOI:** 10.3390/molecules28217265

**Published:** 2023-10-25

**Authors:** Yanping Liu, Xiaonan Wang, Siwei Wang

**Affiliations:** Institute of Plant Protection, Guangdong Academy of Agricultural Sciences, Key Laboratory of Green Prevention and Control on Fruits and Vegetables in South China Ministry of Agriculture and Rural Affairs, Guangdong Provincial Key Laboratory of High Technology for Plant Protection, Guangzhou 510640, China; liuliuyp@tom.com (Y.L.); wangxiaonan@gdaas.cn (X.W.)

**Keywords:** chlorantraniliprole, lychee, risk assessment, residue distribution, terminal residue, dissipation dynamic

## Abstract

This report presents the development of a highly effective method employing high-performance liquid chromatography–tandem mass spectrometry (HPLC-MS/MS) to investigate chlorantraniliprole’s dissipation, risk assessment, and residue distribution in whole lychee fruit and its pulp. Mean recoveries of the samples ranged from 80 to 105%, exhibiting a relative standard deviation (RSD) of below 8%. The limits of quantification (LOQ) for lychee and pulp were determined as 0.001 mg/kg, and half-lives (t_1/2_) ranged from 8.0 to 12.2 days. Terminal residue concentrations in whole litchi and pulp were determined as 0.008–0.45 mg/kg and ≤0.001 mg/kg. These residues were treated twice and thrice at two different dosage levels with pre-harvest intervals (PHIs) of 7, 14, and 21 days. The potential chronic risk posed by chlorantraniliprole to humans was non-negligible, as indicated by the risk quotient (RQ) value not exceeding 1. Therefore, this study provides significant fresh data about the safe application of chlorantraniliprole in the production of lychee, which will help China develop maximum residual limits (MRLs).

## 1. Introduction

Lychee is a prominent economic fruit in the southern areas of China. In particular, China is the globally leading lychee producer, with the highest annual production [1,2]. Lychee is recognized for its abundant nutritional and functional constituents, including vitamins, polysaccharides, and flavonoids [3]. Lychee orchards are vulnerable to various diseases and pests due to the elevated temperature and humidity in the planting environment [4]. These conditions result in a high incidence rate and extended infestations, posing remarkable challenges to effectively managing lychee yield and quality [5]. Chemical control, an integral part of the green prevention system for detrimental organisms in lychee orchards, continues to hold valuable prominence, particularly during disease and pest outbreaks. The expeditious efficacy of chemical pesticides protects lychee’s productivity and quality [6]. Lychee is an essential small horticultural crop in China [7]. However, its cultivation faces challenges due to ineffective pesticide options. Currently, lychee cultivation in China relies on 147 registered pesticide products, which can be categorized into 94 single agents and 53 mixed agents. Among these are 76 fungicide products, 51.7% of the total registered; 45 insecticide products, 30.6%; and 25 plant growth regulator products, 17.0%. Lastly, there is only one herbicide product, making up exactly 0.7% of the total registered pesticide amount. Twenty-seven single and 19 mixed insecticide agents have been officially registered on lychee crops [7]. Among these, 33 pesticide products have been identified to prevent and control *Conopomorpha sinensis* Bradley, 18 lychee stink bugs, and 1 leaf roller [8]. Compared to nearly 80 pests in lychee orchards, few registered insecticides exist. Hence, China must expedite the registration for novel pesticide varieties exhibiting better efficacy, minimal risk, and diverse modes of action specifically targeting lychee.

Chlorantraniliprole, a novel ortho acyl benzamide compound, is a broad-spectrum insecticide developed by DuPont in the United States [9]. It has potent efficacy against various lepidopteron pests infesting vegetable and rice crops. Notable examples include the yellow rice stem borer and leaf folder. The improper application of pesticides on lychees can lead to the contamination of pesticide residues, exceeding the permissible limits in fruit products. Additionally, it can induce toxicity in various environmental organisms, affecting the survival of bees, silkworms, etc. [10,11]. The issues related to quality, safety, and environmental concerns require serious consideration [12]. Hence, the investigation should be directed toward analyzing the residues of pesticides in crops. The surveillance of pesticides in lychee is imperative in maintaining food safety and preventing consumers from possible health hazards [13].

Strategies for analyzing pesticide residues cover sample pre-treatment and instrumental analysis. The implementation of appropriate methods of cleaning is crucial to reduce the risk of matrix interference effects and instrument contamination, thereby ensuring precise quantification. Hence, developing a reliable approach for detecting pesticides in lychee is necessary to reduce potential health hazards and facilitate international trade. The importance of sample pre-treatment cannot be overstated to ensure the validity and reliability of pesticide analysis. Solid-phase extraction (SPE) is a frequently used pre-treatment approach in food analysis [14] and QuEChERS (quick, easy, cheap, effective, rugged, and safe) [15]. Among various methods, samples of cereals, and fruit preparation, the QuEChERS approach has gained significant popularity. The presented method is characterized by its simplicity, stability, sustainability, cost-effectiveness, and high efficiency [16]. LOQ of chlorantraniliprole was determined as 0.001 mg/kg via the QuEChERS method. The detection of chlorantraniliprole residues across multiple matrices is usually involved via HPLC-MS/MS techniques [17] and HPLC [18]. The HPLC-MS/MS has become a proficient technique for identifying and quantifying pesticide residues in recent years. This method has proven highly advantageous due to its exceptional resolution, selectivity, and analytical capabilities, facilitating the detection of a wide array of targeted pesticides across various food matrices. However, to date, there is a lack of scientific investigations related to validating the chlorantraniliprole method in the lychee matrix and assessing its residual properties. 

Chlorantraniliprole has been frequently used in various cultivation, including rice, corn, sugarcane, and cabbage. However, its application on lychee has been relatively infrequent. The dissipation and risk evaluation of chlorantraniliprole in various ecosystems, including paddy ecosystem [19], tomato [20], tobacco plants [21], longan [22], and hawthorn plants, have been previously reported [23]. Paddy leaf and soil residues dissipated within 35–40 and 15–21 days, with a life (t_1/2_) of 3.92–5.07 and 13.33–15.75 days [19]. The dietary risk related to chlorantraniliprole in paddy leaf (green fodder) for cattle indicated that the consumption of chlorantraniliprole poses no significant risk, as evidenced by a hazard index value below 1. The results showed that the soil ecological risk assessment for earthworms (*Eisenia foetida*) and arthropods (*Aphidiusrhopalosiphi*) was determined as RQ ≤ 0.1 [13]. The chlorantraniliprole terminal residues in longan whole fruit at 14 d post-application ranged from 0.06 to 0.29 mg/kg, and the pulp concentration was determined at ≤0.01 mg/kg [22]. The dissipation rate of chlorantraniliprole in hawthorn was observed to conform to first-order kinetics, exhibiting t_1/2_ life ranging from 19 to 26 days. Based on this analysis, chlorantraniliprole in hawthorn does not present an unacceptable risk to the overall health of the general population [23]. Currently, there is a lack of relevant research documenting the impacts of chlorantraniliprole on lychee. Hence, it is necessary to research chlorantraniliprole residual degradation, distribution, and dietary risks on lychees.

The maximum residue limit (MRL) of chlorantraniliprole on lychee in the United States [24], European Union (EU) [25], and Japan [26] was reported as 2 mg/kg, 0.01 mg/kg, 1 mg/kg, respectively. The formation of the MRL for chlorantraniliprole in lychee remains pending in China. Consequently, no investigations have been conducted yet to ascertain the chlorantraniliprole residue in lychee. First, this study attempted to accomplish a highly efficient and sensitive method for detecting and quantifying chlorantraniliprole in lychee samples via the UPLC-MS/MS. Secondly, it assessed chlorantraniliprole’s dissipation kinetics and residues in actual field conditions, including its distribution within the entire lychee fruit, specifically in the pulp. Finally, it examined the safety of chlorantraniliprole in lychee and evaluated its risk by calculating the RQ using the terminal residue analysis results. 

## 2. Results and Discussion

### 2.1. Matrix Effect

The comprehensive influence of components other than analytes on the measured value of the analyte in the sample is defined as the matrix effect (ME). The ME can suppress or increase the analyte response, which could lead to deviation in the accuracy, precision, and sensitivity of the quantitative method. The ME was usually calculated using the equation ME = (slope of the matrix-matched standard/slope of the solvent standard − 1) × 100%. When the|ME| ≤ 20%, it indicates that the matrix does not exist; 20% ≤ |ME| ≤ 50% indicates a medium matrix effect; and |ME| ≥ 50% indicates a strong matrix effect [27]. As shown in Table 1, there was a medium ME in the whole lychee (|ME| = 46.1%), and there was no ME in the pulp. In this study, we employed an external matrix-matched standard curve to remove the impact of matrix effects.

### 2.2. Method Verification

The analytical method was validated by the following parameters: linearity, recovery, precision, and accuracy. Recovery rates were calculated to assess precision and accuracy. Matrix-matched (0.001–0.5 mg/L values) samples were used to determine the linearity of whole lychee and pulp samples. Both of the correlation coefficients (r^2^) exceeded 0.999. The precision and accuracy of the experimental measurements were determined via the implementation of recovery studies. In this study, five replicate samples were analyzed at three distinct concentrations of chlorantraniliprole, as shown in Table 1.

**Figure 1:**
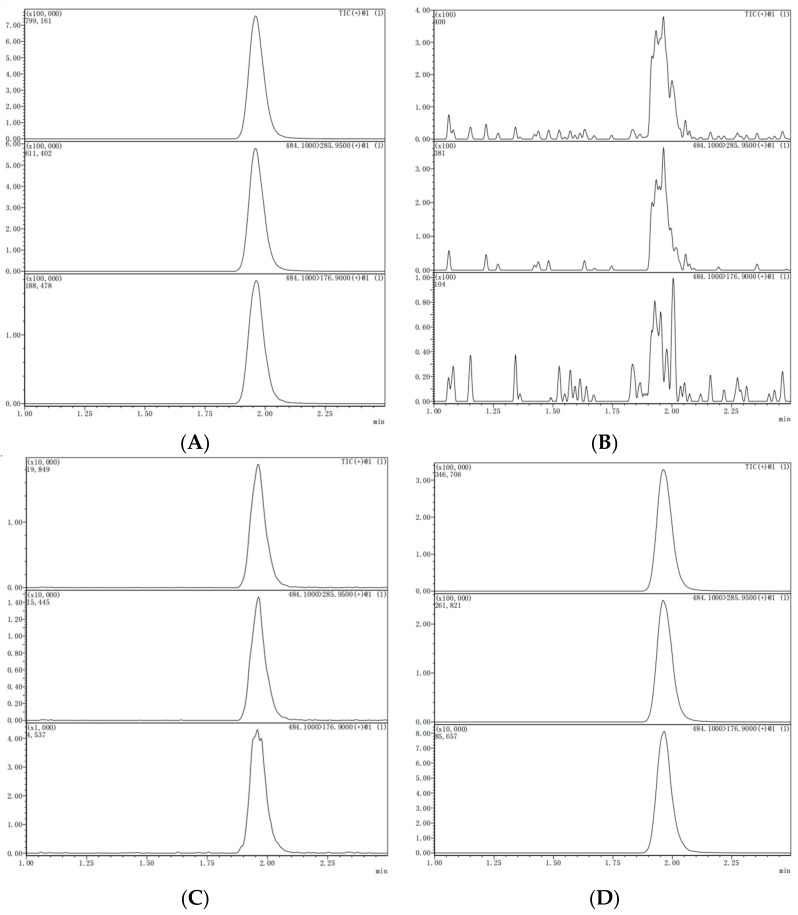
Chromatogram of chlorantraniliprole standard, blank, spiked sample and real sample (Lateral axis is time, and longitudinal axis is response intensity). (**A**) chlorantraniliprole standard (0.01 mg/kg); (**B**) lychee CK; (**C**) spiked lychee sample (0.01 mg/kg); (**D**) lychee real sample.

The mean recoveries for all concentration levels were within the satisfactory range of 80–105%, with RSDs from 4–7% in whole lychee samples. Similarly, the mean recoveries ranged from 80 to 99% in pulp samples, with RSDs of 5–9%. These findings demonstrate that this method conforms to the standard validation criteria for analyzing pesticide residues. The LOQ for chlorantraniliprole in whole lychee and pulp was 0.001 mg/kg. The experimental findings demonstrated that the employed technique yielded results that dropped within the acceptable range, exhibiting high validity and reliability. The UPLC-MS/MS chromatograms for the spiked and blank samples are shown in Figure 1.

### 2.3. Dissipation of Chlorantraniliprole in Lychee

**Figure 2:**
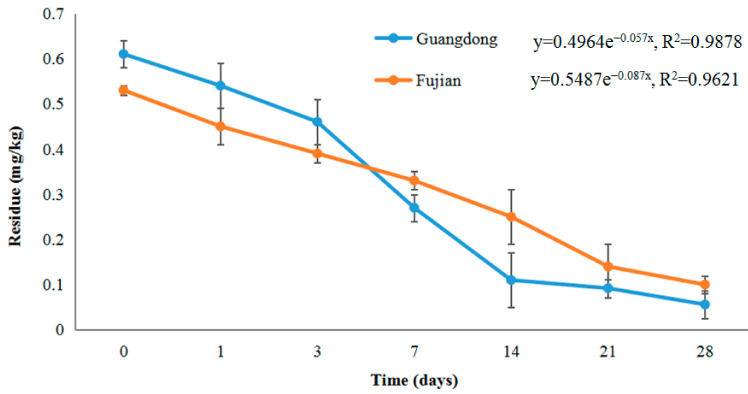
The dissipation pattern of chlorantraniliprole in lychee (lateral axis is collected sample time, and the longitudinal axis is residues).

Figure 2 illustrates the dissipation pattern of chlorantraniliprole in lychee in open-field conditions. The findings demonstrated that the amount of chlorantraniliprole in lychee fruit from the Guangdong region is comparable to that found in lychee fruit from the Fujian region. Precisely, the initial deposit of chlorantraniliprole was measured to be 0.53 mg/kg in the Fujian and 0.61 mg/kg in the Guangdong regions. The amount of chlorantraniliprole in lychee fruit treated in Guangdong exhibited a significant decrease in 14 days following the application, ultimately reaching a level of 0.011 mg/kg. This reduction corresponded to a substantial loss of 82%. The degradation rate of chlorantraniliprole was rapid, resulting in less than 0.056 mg/kg, with a considerable reduction of 91% after a 28-day post-application. Following a 28-day application in Fujian, the recorded concentration of chlorantraniliprole residue was determined as 0.10 mg/kg. The climatic conditions at the two experimental sites exhibited variations; however, the degradation t_1/2_ life estimated by the regression equation was determined at 8.0 and 12.2 days in Guangdong and Fujian. 

The dissipation of residues can be assigned to various variables, including pH, sunlight, moisture, and temperature. Additionally, it has been documented by several researchers that the decline of residues can be influenced by the growth dilution factor and the choice of crop varieties [28]. The findings indicated that harvesting can be performed without safety concerns, primarily 14 days after applying the suggested chlorantraniliprole dose. 

### 2.4. Terminal Residues of Chlorantraniliprole in Lychee

The information summarized in Table 2 presents the final residue levels of chlorantraniliprole found in both whole lychee and pulp samples obtained from the treated plots. The terminal residue concentrations of chlorantraniliprole in lychee at 7, 14, and 21 days after the final application were examined for whole lychee. The residues of chlorantraniliprole resulting from two and three applications at the recommended 50 mg/kg dosage were noted. The observed residue levels were from 0.042 to 0.25 mg/kg, 0.019 to 0.19 mg/kg, and 0.008 to 0.14 mg/kg, considering PHIs of 7, 14, and 21 days. The observed trend in the concentrations under different PHIs indicated decreased residues as the PHIs increased. The effects of multiple applications of chlorantraniliprole at 1.5 times the suggested dosage (75 mg/kg) were investigated at 7, 14, and 21 days. The concentrations of emamectin benzoate were determined to be in the range of 0.061–0.45, 0.026–0.31, and 0.012–0.25 mg/kg. Positive correlations were observed among the residues and application times, indicating that a prolonged harvest interval was linked to reduced residue levels.

Compared to the pulp, the residues in the entire lychee fruit were considerably higher. Furthermore, at 0.001 mg/kg, all terminal residues found in the pulp were below the LOQ. Most residues are hypothesized to exhibit a higher concentration in the peel. Applying chlorantraniliprole SC onto the peel showed no direct exposure of the pulp to the pesticide. In contrast, the fat-soluble insecticide chlorantraniliprole has a Kow logP (2.76, pH 7) (The e-Pesticide Handbook, Version 3.0).

Consequently, it is expected to exhibit an increased capacity for absorption in the peel. Therefore, the levels of residues that infiltrated into the pulp were found to be considerably low. According to available reports, the residual distribution pattern of most pesticides generally conforms to the observation that the residual quantity in the whole fruit far exceeds that in the pulp [29,30]. However, pesticide residues, including carbendazim, methomyl, and thiabendazole, have been much more significant in the pulp than in the rest of the fruit. Pesticide residues in fruits can be categorized into two distinct mechanisms. The first mechanism involves the adhesion of pesticides to the fruit’s surface. The second mechanism entails the internal circulation of endogenous pesticides within the plant body, leading to their distribution in various plant parts. The surface of the fruit is typically covered by a wax coating, exhibiting essential hydrophobic properties that effectively impede the infiltration of hydrophilic contaminants into the fruit’s internal structure. Pesticide residues in fruits predominantly originate from the translocation of branches and leaves, subsequently accumulating within the fruit’s flesh upon entry via the stem. The distribution of pesticides in fruits is affected by multiple factors, including the characteristics of the pesticides, the surroundings, fruit properties, application approaches, and agronomic measures [31].

According to the results, the number of chlorantraniliprole residues in lychee fruit exhibited a positive relationship with the application dosage. However, the observed variations in residue levels across the six regions were deemed statistically insignificant, and the temporal period of crop harvesting considerably impacted the terminal residues. All lychee samples exhibited residue levels 21 days after the chlorantraniliprole application that were either considerably below or equivalent to the acceptable quantity, 0.14 mg/kg and 0.25 mg/kg at 1.5 times the prescribed amount, respectively. It indicates that the amount of terminal residues is elevated when the PHI is shorter.

Due to the lack of an MRL of chlorantraniliprole on lychee in China, the residual data related to the PHI of 14 days for chlorantraniliprole on lychee were examined. The analysis revealed a median residue value of 0.115 mg/kg, whereas the highest residue reached 0.31 mg/kg. The maximum residue limit of chlorantraniliprole on lychee is recommended to be 1 mg/kg. The recommended application for preventing and controlling lychee stem borers on lychee is a 5% chlorantraniliprole SC, and the maximum acceptable dosage is 75 mg/kg, with a maximum of 3 applications with a PHI of 14 days.

### 2.5. Risk Assessment

Risk assessment of chlorantraniliprole in lychee has been investigated on the typical food consumption patterns of the Chinese population [32]. Exposure to chlorantraniliprole via lychee fruit was examined using the RQ method. *ADI* values (*bw* = 2 mg/kg) were obtained from the JMPR report for risk assessment of chlorantraniliprole residues in lychee pulp samples [33]. Outcomes for intake estimations that fell below the LOQ were considered LOQ values. In entire lychees, the *STMR* of chlorantraniliprole was found to be 0.001 mg/kg.

The potential hazard with prolonged use of chlorantraniliprole was evaluated in both genders, within the age groups of 2–4, 18–30, and 60–70 years, with a risk percentage from 0.0015% to 0.0147%. Notably, these values were found to be lower than 100%. The chlorantraniliprole at 50 mg/kg and 75 mg/kg, along with two or three applications and a PHI of 7, 14, and 21 days, was also considered (Table 3). These findings demonstrated that applying chlorantraniliprole on lychee does not exhibit any visible chronic dietary risk to Chinese consumers.

### 2.6. Discussion

The residues of chlorantraniliprole in lychee are closely related to some factors such as application concentration, application frequency, pre-harvest intervals, and field climate conditions. The residues increased as the concentration and frequency of application increased, and the residues decreased as the pre-harvest intervals increased. This rule is consistent with the residual results on longan [22]. The residues of chlorantraniliprole in pulp are lower than that in the whole lychee, which is related to factors such as the octanol water partition coefficient and water solubility. Pesticide residues (metalaxin, pyrethroid, chlorpyrifos, imidacloprid, triazophos, cypermethrin) in lychee are mainly distributed in the peel, and the pesticides will not migrate from the peel to the pulp when samples were stored under 4 °C, 1 °C, −18 °C [34]. Some studies have also shown that by detecting the pulp and peel of lychee samples from the market, it was found that the detection rate of pesticides (including chlorantraniliprole) in the whole fruit of lychee was 100%, and the pulp was 70%. When measured by the ratio of the maximum residue in lychee pulp to the whole fruit, most or even the vast majority of pesticide residues were present on the peel [35]. The above report is consistent with the results of this study.

Lychee is a minor crop, and many pesticides are not registered on lychee. Hence, the usage method of unregistered pesticides on lychee can only refer to the usage standards of other registered crops and is not suitable for the actual production of lychee [35]. In addition, there are still shortcomings in the formulation of pesticide residue limits on lychees in China, and research on the usage technique and residue limits of pesticides on lychees urgently needs to be strengthened.

## 3. Material and Methods

### 3.1. Chemicals and Reagents

Chlorantraniliprole (97.8%) standard was procured from Dr. Ehrenstorfer GmbH (Augsburg, Germany). The DuPont Company (Wilmington, DE, USA) supplied a suspension agent (SC) with a 5% chlorantraniliprole concentration. The acetonitrile, methanol, and HPLC grade solutions were acquired from Thermo Fisher Scientific Inc. (Waltham, MA, USA). From Fisher (Pittsburgh, PA, USA), we obtained chromatographically pure acetonitrile (MeCN). Sinopharm Chemical Reagents Co., Ltd. (Shanghai, China) supplied analytical grade anhydrous magnesium sulfate (MgSO_4_) and sodium chloride (NaCl). The Aladdin Industrial Corporation in Shanghai, China, provided HPLC-grade formic acid. Agela Technologies Inc. (Tianjin, China) supplied the sorbent materials, including secondary amine (PSA). Millipore’s Simplicity UV water-purification system (Bedford, MA, USA) was used to purify the water.

Stock solutions of each standard pesticide were formulated using acetonitrile as the solvent at 1000 mg L^−1^ and kept at −20 °C. Next, the working solutions utilized in the calibration and fortification were prepared fresh by diluting the stock solution using acetonitrile. The solvent calibration solutions were prepared via mixing in acetonitrile. Blank extracts from every matrix were combined with the stocks to produce the matrix-matched solutions. Before being used, all produced solutions were maintained at 4 °C.

### 3.2. Field Trials and Sample Preparation

The field experiments involved residue dynamic and terminal residue analysis. These experiments were conducted at 6 different sites, including Hainan Province (Haikou City), Fujian Province (Putian City), Yunnan Province (Baoshan City), Guangxi Province (Nannign City), and Guangdong Province (Maoming and Guangzhou City), in 2018. The People’s Republic of China’s Ministry of Agriculture released a “Guideline on Pesticide Residue Trials” (NY/T 788-2004) that was followed in the present study according to the directions available in pesticide labels for the design and execution of the experiment.

The terminal residue experiment was conducted during a supervised field trial, in which the recommended 50 mg/kg dosage was administered. A maximum of 75 mg/kg (1.5 times the recommended dose) was also given. A 5% SC containing chlorantraniliprole was administered 2 or 3 times, with a 7-day interval during each application, with low and high concentrations. Each experimental plot consisted of a pair of lychee trees. The experimental design consisted of three replicate plots for each treatment and one control. Respective samples (2 kg lychees) were collected at preharvest intervals (PHIs) of 7, 14, and 21 days. The samples were obtained from various points within each plot following the completion of the last spraying. The experimental samples for the terminal residue analysis comprised the whole lychee fruit, including the pulp. The lychee samples were split into smaller aliquots using the quartering method before homogenization for subsequent analysis.

To examine the dissipation of chlorantraniliprole in lychee, a 5% chlorantraniliprole SC was prepared by dissolving chlorantraniliprole in water. The lychee plots were treated with 100 mg/kg of SC when the lychee exceeded 50% of fully ripe fruits. A comparative analysis was conducted on a plot of identical dimensions, wherein no application of chlorantraniliprole was administered simultaneously. Samples of lychee were taken from every plot to investigate dissipation at 2 h, 48 h, 3 days, 7 days, 10 days, 14 days, and 21 days post-application. All specimens were preserved at a temperature of −20 °C for later examination.

Each sample comprised a minimum of 2 kg of lychee. Before processing, foreign material such as desiccated foliage and dirt particles on the fruit’s surface was removed. Using a food processor, the collected samples were promptly homogenized without washing or peeling. The homogenized samples were transferred into the hermetically sealed polyethylene containers and freezed at −20 °C. These frozen samples were transported to the laboratory in a sealed container with sufficient ice and promptly maintained at freezing temperature until analysis. The samples were examined within a month after homogenization.

### 3.3. Extraction and Cleanup Procedures

The whole lychee or lychee pulp was weighed (10 g) and added into a centrifuge tube (50 mL). Subsequently, acetonitrile (20 mL) was added to the tube, and the contents were vigorously mixed with a vortex mixer at 15,000 rpm/min for 1 min. After adding 5 g of NaCl, the samples underwent vigorous shaking for 1 min and were centrifuged at 3800 rpm for 5 min. The resultant supernatant (4 mL extract) was evaporated via a rotary vacuum evaporator at 40 °C and mixed in 2 mL methanol for purification.

The methanol-fixed solution (2 mL) was transferred into a 5 mL tube containing 150 mg of anhydrous MgSO_4_ and 50 mg of PSA to promote the cleanup procedure. Following a 1 min vigorous shaking, the samples were centrifugated at 5000 rpm for 5 min. The resulting supernatants were then filtered via a nylon syringe filter (0.22 μm) and transferred to an autosampler vial for subsequent analysis via HPLC-MS/MS.

### 3.4. Instrumentation and UPLC-MS/MS Analytical Conditions

Chlorantraniliprole was extracted using the Shimadzu LC-20A HPLC system equipped with a Shimadzu Shim-pack GIST-HP C18 column (100 × 2.1 mm, with a stationary film thickness of 2.5 μm) and maintained at a column oven at 35 °C. The separation was conducted using mobile phases A (0.1% aqueous formic acid) and B (MeCN) (*v*/*v*, 60/40). The flow rate was maintained at a value of 0.3 mL/min. Injecting a 1 uL sample with an autosampler, separation, and equilibrium were attained in 9 min.

A triple quadrupole Shimadzu 8045 mass spectrometer (Shimadzu; Kyoto, Japan) was used to analyze the samples with positive ion mode electrospray ionization (ESI+). The instrument parameters were set as follows: heating block temperature = 400 °C, desolvation line (DL) = 250 °C, and oven temperature = 350 °C. Nitrogen was used as the collision gas and nebulizer, and chlorantraniliprole was evaluated via multiple reaction monitoring. The numbers of the precursor and product ions of chlorantraniliprole were 484.1, 285.95, and 176.7, and their collision energies were −14 and −48 eV, correspondingly.

### 3.5. Methodological Validation

The precision and dependability of the developed methods were examined using LOQ values, linear equations, and recovery rate (RR). The prepared standards demonstrated a linear relationship within the 0.001 to 0.5 mg L^−1^ concentration range. The RR values were achieved by employing blank food samples artificially contaminated with chlorantraniliprole at 0.001, 0.01, or 0.1 mg kg^−1^. Each concentration was replicated five times to ensure statistical reliability. This study’s methodological precision assessment used RSDs. The lowest detected elevated level in the specified matrix was used to calculate the LOQ for each compound (SANTE/11813/2017).

### 3.6. Statistical Analysis

The dissipation kinetics of chlorantraniliprole in lychee were investigated by constructing a plot of residue concentration against time. The equations of best-fit curves were determined via the maximum squares of the correlation coefficient. Confirmation of the first-order kinetics was further verified via graphical analysis using the following equation: C_t_ = C_0_e^−kt^, where C_t_ indicates the quantity of pesticide residue at time t, k is the dissipation degradation rate constant, and C_0_ reflects the initial concentration after application. The t_1/2_ life of dissipation (t_1/2_) was determined as ln2/k. All values are expressed as means ± standard deviation (SD) of 5 replicates.

The risk assessment and dietary exposure were determined using the RQ method to apply chlorantraniliprole safely. RQ value above 1 indicates an unacceptable pesticide risk, while below 1 shows a minimal risk to humans. Chronic dietary exposure assessment [36] evaluated the possible hazards related to long-term consumption. This assessment involved calculating exposure levels by considering the median concentrations of pesticide residues on longan fruit and the acceptable daily intake (*ADI*, mg/kg/d). The chronic risk quotient, referred to as *ADI%*, was determined using the following formula:

*ADI%* = (*STMR* × FI)/(*ADI* × *bw*) × 100%,

where *bw* (kg) represents the mean body weight, FI (kg/day) is the dietary reference intake for a certain kind of food used to examine nutrient intakes of the healthy Chinese population, and *STMR* (mg/kg) is the supervised trials median residues. According to the China Health and Nutrition Survey report, the *bw* observed among adults in China was recorded as 63 kg. 

## 4. Conclusions

Chlorantraniliprole residue was quantified in the whole lychee fruit and its pulp using a combination of QuEChERS and HPLC-MS/MS approaches. To verify the secure application of chlorantraniliprole, its dissipation, terminal residue, distribution, and risk factors were evaluated, and it revealed that chlorantraniliprole dissipation in lychee occurred rapidly, with an estimated t_1/2_ of 8.0 to 12.2 days. This dissipation followed the laws of first-order kinetics. The terminal residues in the whole lychee were determined to be substantially higher compared to the pulp. The residues in the pulp were determined to be consistently under the limit of quantification of 0.001 mg/kg. The dietary health risks caused by chlorantraniliprole residues in the pulp at the recommended dosage were determined non-negligible, as indicated by the chronic risk quotient value not exceeding 1. Hence, based on the above research data, the reasonable suggestion of using chlorantraniliprole on lychee, i.e., 5% chlorantraniliprole SC, can be used to prevent and control *Conopomorpha sinensis* Bradley and looper of lychee, with a maximum dosage of 75 mg/kg and a maximum of three applications. The safe pre-harvest interval is 14 days. The recommended MRL for chlorantraniliprole on lychee is 1 mg/kg. At present, China has not yet established the MRL value of chlorantraniliprole on lychee. This study has clarified the degradation dynamics and final residue levels of chlorantraniliprole on lychee, which can provide basic evaluation data for the registration and use of this pesticide on lychee in China.

## Figures and Tables

**Table 1:** Performance characteristics of the method for chlorantraniliprole in the whole lychee and pulp.

Matrix	Fortified Level ^a^ (mg/kg)	Average Recovery ^b^ (%, *n* = 5)	RSD ^c^ (%)	Correlation Coefficient	|ME| (%)	LOQ (mg/kg)
lychee	0.001	86	5	0.9992	46.1	0.001
	0.01	89	4			
	0.1	95	7			
pulp	0.001	87	8	0.9997	15.4	0.001
	0.01	88	6			
	0.1	94	5			

^a^ The standard fungicide was spiked before the sample grinding. ^b^ The recovery was calculated using the formula: Recovery = *C_d_/C_s_* × 100%, where *C_d_* represents the detected concentration and *C_s_* represents the spiked concentration. Results were expressed as mean ± standard deviation (SD) with 95% confidence intervals.^c^ Mean value of five determinations.

**Table 2:** Terminal residues of chlorantraniliprole in whole lychee.

Application Dosage (mg·kg^−1^)	Times	Mean, Median ^a^, and HR ^b^ in the Whole Fruit at Different PHI ^c^ (mg·kg^−1^)		
		PHI 7 d	PHI 14 d	PHI 21 d
50	2	0.11/0.11/0.21	0.07/0.06/0.16	0.03/0.02/0.084
	3	0.14/0.14/0.25	0.09/0.09/0.19	0.06/0.05/0.14
75	2	0.18/0.17/0.35	0.13/0.12/0.29	0.08/0.09/0.15
	3	0.22/0.22/0.45	0.16/0.15/0.31	0.10/0.09/0.25

Note: ^a^ Mean supervised trials median residue. ^b^ Mean highest residue. ^c^ Mean pre-harvest interval. Terminal residues of chlorantraniliprole in pulp were all below LOQ. HR is the abbreviation of high residue. PHI is the abbreviation of pre-harvest interval.

**Table 3:** The risk evaluation of chlorantraniliprole in lychee.

Application Dosage (mg·kg^−1^)	Times	PHI (Days)	*ADI* (mg·kg^−1^·d)	*STMR* (mg·kg^−1^)	%*ADI*					
					2~4 Male	2~4 Female	18~30 Male	18~30 Female	60~70 Male	60~70 Female
50	2	7	2	0.01	0.0015	0.0107	0.0021	0.0145	0.0055	0.0117
		14		0.01	0.0016	0.0106	0.0021	0.0143	0.0057	0.0112
		21		0.01	0.0015	0.0106	0.0022	0.0138	0.0055	0.0117
	3	7		0.01	0.0016	0.0107	0.0021	0.0146	0.0056	0.0115
		14		0.01	0.0016	0.0106	0.0021	0.0144	0.0054	0.0116
		21		0.01	0.0015	0.0106	0.0021	0.0144	0.0054	0.0114
75	2	7		0.01	0.0015	0.0107	0.0021	0.0144	0.0055	0.0115
		14		0.01	0.0016	0.0107	0.0021	0.0146	0.0055	0.0115
		21		0.01	0.0016	0.0107	0.0021	0.0147	0.0055	0.0117
	3	7		0.01	0.0016	0.0107	0.0021	0.0146	0.0055	0.0115
		14		0.01	0.0015	0.0107	0.0021	0.0144	0.0055	0.0117
		21		0.01	0.0016	0.0107	0.0021	0.0147	0.0055	0.0115

## Data Availability

Not applicable.
